# A Study on Bus Passenger Boarding and Alighting Detection and Recognition Based on Video Images and YOLO Algorithm

**DOI:** 10.3390/s26051418

**Published:** 2026-02-24

**Authors:** Wei Xu, Yushan Zhao, Xiaodong Du, Haoyang Ji, Lei Xing

**Affiliations:** College of Transportation, Qingdao Campus, Shandong University of Science and Technology, Qingdao 266590, China; xuwei972@163.com (W.X.); 202582160031@sdust.edu.cn (Y.Z.); 202383160020@sdust.edu.cn (X.D.); 202483160044@sdust.edu.cn (H.J.)

**Keywords:** deep learning, object tracking, object detection, bus OD

## Abstract

Public transportation is the core of easing urban traffic congestion, reducing pollution and advancing smart city transportation intellectualization. Its refined operation relies heavily on accurate, real-time passenger origin–destination (OD) data. However, traditional manual surveys are costly with low sampling rates, while smart card big data lacks alighting information and has deviations, failing to reflect real travel behaviors and becoming a bottleneck for intelligent public transportation development. To address this, this paper proposes a bus passenger boarding/alighting detection and recognition study based on video images and the YOLO algorithm. Aiming at traditional YOLO’s shortcomings in on-vehicle scenarios (insufficient feature extraction, inefficient feature fusion, slow convergence), the baseline YOLOv8n is improved for bus scenarios’ high-density, high-occlusion and variable-target scales: (1) DAC2f structure (deformable attention + C2f) captures occluded passengers’ core features and suppresses background interference; (2) SWD-PAN enables bidirectional cross-scale feature interaction to adapt to scale differences; and (3) WIoUv3 balances sample weights for small targets and non-standard posture passengers. Experiments show that precision, recall and mAP increase by 3.68%, 5.12% and 6.26%, respectively, meeting real-time requirements. The improved YOLOv8 is deeply integrated with DeepSORT to enhance tracking stability. Tests show that MOTA reaches 31.24% (2.6% higher than YOLOv8n, 16.4% higher than YOLO-X) and MOTP reaches 88.06%, solving trajectory breakage and ID switching. This addresses traditional OD data collection pain points, providing technical support for intelligent public transportation refined management and smart city transportation optimization.

## 1. Introduction

With China’s accelerating urbanization and deepening new urbanization strategy, urban population agglomeration is prominent, and residents’ travel features total growth, diverse structures, and temporal–spatial concentration. Ultra-large and megacities often see daily public transport passenger volumes exceeding 10 million, with peak-hour core road congestion, undermining travel efficiency, exacerbating energy consumption and pollution, and becoming a major bottleneck for urban sustainable development. As a green, low-carbon, efficient, and intensive mode, public transport is key to easing traffic pressure and reducing carbon emissions, as well as being a core carrier for “on-demand mobility” systems. Recent public transport network expansion has made smart public transport’s refined operation and scientific planning increasingly dependent on accurate, real-time, full-dimensional passenger flow data.

However, current data collection methods have significant limitations. Traditional statistics rely on two approaches: (1) Labor-intensive, costly on-board/household travel surveys, constrained by a 5–10% low sampling rate and narrow periods, failing to fully reflect passenger flow characteristics and special scenario patterns; (2) electronic payment data, lacking alighting information and excluding cardless/fare-exempt groups, causing deviations. These issues prevent operators from grasping the temporal–spatial distribution and OD correlations, hindering service quality and efficiency. Thus, accurate boarding/alighting station identification via technological innovation and a complete data chain is urgently needed for intelligent public transport.

Meanwhile, “Safe Cities” and “Intelligent Transportation” initiatives have achieved full on-board surveillance camera coverage, providing a new technical path. Cameras capture real-time passenger data (counts, positions, trajectories, boarding/alighting behaviors), enabling automated, high-precision collection via computer vision. Seizing this opportunity, this paper optimizes video image analysis and the YOLO algorithm to conduct bus passenger boarding/alighting detection research, aiming to develop a rapid method integrating target detection, multi-target tracking, and behavior extraction to break traditional bottlenecks.

Nevertheless, bus-scenario complexity poses unique challenges: narrow enclosed carriages with peak-hour density and frequent occlusions, unstable lighting (backlighting, sudden changes, reflections), and dynamic boarding/alighting with rapid target scale changes and background switching. Traditional algorithms suffer from insufficient feature extraction, poor fusion, and slow loss function convergence, failing to adapt to bus scenario needs.

Against this backdrop, this paper proposes a study on bus passenger boarding/alighting detection based on video images and the YOLO algorithm, improving DeepSORT’s detector component to enhance performance in dense and occluded scenarios. Compared with existing methods, the core scientific contributions are clearly defined as follows:

1. A DAC2f structure is proposed, which deeply couples the dynamic sampling characteristics of the deformable attention mechanism with the feature fusion ability of the C2f module, adaptively captures the core features of occluded passengers, suppresses background interferences such as seat armrests, and solves the key problem of blurred target features in dense occlusion scenarios. Its innovation lies in combining adaptive sampling of target regions with local feature enhancement, breaking through the global fixed processing paradigm of traditional attention mechanisms.
2. A SWD-PAN bidirectional feature pyramid network is constructed, which introduces a weighted adaptive fusion mechanism and a structural simplification strategy, breaks the one-way information transmission barrier of traditional FPN, realizes bidirectional iterative interaction of cross-scale features, adapts to the scale differences between near and far passengers in the door area, and provides a more efficient feature interaction scheme for multi-scale target detection.
3. The superiority of the WIoUv3 loss function in the on-board scenario is verified, and the dynamic non-monotonic focusing mechanism is used to balance the training weights of extreme samples and medium-quality samples, adapting to the detection needs of small targets and passengers in non-standard postures in the bus scenario, establishing a matching relationship between the loss function design and the target characteristics of the bus scenario, and enriching the loss optimization theory of dense target detection.
4. The deep integration of the improved YOLOv8 and DeepSORT is completed, the interactive logic of the detection-tracking module is optimized, the tracking stability in scenarios with dense passenger flow and occlusion is improved, and the problems of trajectory breakage and ID switching in the bus boarding and alighting scenario are effectively solved.

Through these innovations, the improved algorithm achieves certain gains in precision, recall, and mAP compared with baseline YOLOv8n. This breakthrough reduces reliance on manual labor, cuts operational costs, and provides real-time, accurate OD data for intelligent scheduling, offering reliable technical support for smart public transport’s refined management and smart city transportation optimization, with broad application prospects and long-term promotion value.

## 2. Related Work

### 2.1. Pedestrian Target Detection

The introduction of Convolutional Neural Networks (CNN) by Krizhevsky et al. [1] marked a pivotal shift in target detection, transitioning the field from a reliance on handcrafted features to deep learning and catalyzing the evolution of single-stage and two-stage architectures. Prior to this shift, traditional methods depended on handcrafted features and classifiers, with notable contributions including HOG features combined with SVM classifiers [2] and the Deformable Part Model (DPM) [3], which advanced pedestrian detection through sophisticated feature design.

Current research in pedestrian detection focuses on complex scene detection, small-target detection, and high real-time performance. Algorithms are broadly categorized into two-stage and single-stage architectures. Two-stage detectors, such as R-CNN, Fast R-CNN, Faster R-CNN, and Mask R-CNN [4,5,6,7], prioritize accuracy through region proposal mechanisms. In contrast, single-stage detectors like YOLO, SSD, and RetinaNet [8,9,10] emphasize speed, with ongoing iterations such as YOLOv5 and YOLOv7 further balancing accuracy and efficiency through network optimization and enhanced feature fusion.

Despite these advances, both categories face critical bottlenecks: two-stage methods are computationally expensive with slow inference, limiting real-time use; single-stage detectors, though faster, lack accuracy in complex scenes and for small targets. Both also encounter edge deployment hurdles due to model complexity and large parameters.

To address these issues, researchers have pursued algorithm optimization, scene adaptation, and model lightweighting. For two-stage algorithms, efficiency has been improved through integration with single-stage modules and lightweight networks. For single-stage methods, enhancements include attention mechanisms [11], data augmentation for adverse conditions [12], and refined feature fusion for small targets [13]. Lightweight designs, such as the Stem module in YOLOv5 [14], have also been introduced to facilitate edge deployment. Further optimizations involve attention mechanisms for feature focus, network modifications, and improved detection mechanisms like Soft-NMS [15], alongside data augmentation techniques to enhance model robustness.

In summary, YOLO-based algorithms balance speed, accuracy, and lightweighting, despite challenges in complex scenes and small-target detection. This study adopts the YOLO series as its core technical framework, focusing on optimizing feature extraction, enhancing small-target detection accuracy, and enabling lightweight model deployment to advance practical applications in pedestrian target detection.

### 2.2. Pedestrian Multi-Target Tracking

The evolution of visual multi-target tracking has progressed from early optical flow methods—burdened by high computational cost and limited real-time adaptability—to the more efficient Kalman filter [16], albeit with inherent constraints for non-linear motion. Current methodologies are broadly categorized into three paradigms: classical algorithms (including Kalman filtering and Meanshift/Camshift [17,18]), correlation filter-based methods, and deep learning-based approaches. Represented by MOSSE, CSK, and KCF [19,20,21], correlation filter methods enhanced robustness through techniques like regularization and ridge regression, performing well in moderately complex scenes. The advent of deep learning, leveraging CNNs, Siamese networks, and Transformers for autonomous feature learning, has since dominated the field due to superior accuracy and adaptability.

The development of correlation filters began with MOSSE [19] for fast position prediction, evolved with CSK’s regularization [20], and advanced with KCF’s ridge regression [21] and DSST’s scale adaptation [22]. Deep learning brought a paradigm shift: the HCF algorithm merged deep features with correlation filters in 2015, fully convolutional Siamese networks reframed tracking as image matching in 2016, and DeepSORT [23] significantly reduced identity switches in 2017 through deep appearance descriptors and cascade matching.

Despite these advances, practical challenges remain. Classical and correlation-filter methods struggle with real-time demands, scale variation, and severe occlusion. To address these, recent research has proposed targeted solutions. For instance, Cheng et al. [24] optimized feature fusion for cross-modal RGB-T tracking, Zhao et al. [25] applied ByteTrack to zebrafish tracking for better occlusion handling, and Han et al. [26] combined improved YOLOX with multi-level data association for stable pedestrian tracking.

Further optimizations focus on core components: introducing adaptive noise coefficients to improve filter convergence [27], employing hybrid attention mechanisms to suppress background interference [28], and fusing instance segmentation with robust association methods for marker-free multi-object tracking [29].

### 2.3. Dense Object Detection

Dense object detection, a core computer vision task, identifies and localizes targets in dense, multi-scale, or occluded scenes. Early detectors fall into two categories: two-stage and single-stage. Single-stage ones are prominent for streamlined workflows and edge-device compatibility, treating detection as dense classification and bounding box regression. However, they face quality assessment inconsistencies and lack precise guidance, while conventional models like YOLO and Faster R-CNN often miss detections in crowded scenarios.

Current research has crystallized into six main technical directions: (1) loss function improvements, e.g., GFocal [30], which extends Focal Loss for joint representation optimization; (2) Transformer-driven global matching networks like GMTNet [31], leveraging sliding windows and dynamic matching; (3) scene context-guided frameworks such as SCDNet [32], designed for remote sensing tiny-object detection; (4) enhanced YOLO algorithms like DCYOLO [33], optimized for congested urban streets; (5) dual-network architectures, e.g., S-Net+D-Net [34], improving saliency detection via distraction diagnosis; and (6) cross-split strategies such as Cross-splitNet [35], enabling adaptive feature extraction based on target density.

The field’s evolution has progressed from discrete to continuous loss functions, from single-scale to multi-scale feature fusion, from fixed to dynamic sample matching for handling proposal redundancy, and from neglecting to leveraging scene context to overcome tiny-object detection limits. Notably, NanoDet, building upon GFocal, improves AP by 1.8 over YOLOv4-Tiny while doubling speed and reducing model size sixfold [30].

Despite these advances, practical challenges remain, including complex backgrounds, occlusion, target adhesion in industrial settings, tiny objects in remote sensing, and scale variation/overlap in urban and natural scenes, leading to high miss rates, slow convergence, and poor generalization in traditional models.

In response, targeted solutions have emerged: GMTNet mitigates background interference via global dynamic matching [31]; SCDNet enhances tiny-object detection with a scene classification sub-network [32]; DCYOLO boosts AP by 9.1% in urban scenes using a difference-sensitive network and SIoU loss [33]; and Cross-splitNet improves mAP by 1.9% on COCO through adaptive feature extraction [35].

Current optimization strategies focus on three core methodologies: refining the representation layer by integrating quality estimation into classification; enhancing feature fusion through cross-scale and context-aware mechanisms; and innovating training with dynamic sample matching and adaptive adjustments. These advances address both domain-specific challenges and propel the general advancement of dense object detection.

### 2.4. Summary of Research Status at Home and Abroad

Deep learning is widely adopted in intelligent transportation systems, especially for pedestrian recognition and tracking, learning inherent patterns from traffic data via iterative training to enable cognitive decision-making. Research on video-based public transport passenger flow OD analysis using YOLO algorithms mainly includes passenger detection and tracking. Conventional pedestrian detection relies on candidate region feature extraction and classification, while YOLO series methods perform well but have limitations like false positives and missed detections in complex environments, prompting this paper to propose an improved YOLOv8-based detection algorithm. Target tracking reconstructs motion trajectories from sequential data, with common methods including filtering-based and deep learning-based ones; DeepSORT, built on SORT, uses detection results but has issues like large parameters and poor re-identification under occlusion, so an enhanced version is introduced. Dense object detection has advanced through iterative optimization, shifting from traditional to deep learning-driven architectures like YOLO and Transformers. Current research focuses on improving YOLOv8-based detection and DeepSORT tracking to overcome detection errors and occlusion handling issues, with future advances emphasizing scene adaptability via lightweight design, refined feature fusion, and tighter tracking-detection integration for higher accuracy and robustness.

## 3. Method

### 3.1. Detector

#### 3.1.1. DAC2f Structure

This paper designs a deep fusion of the deformable attention mechanism (DAttention, DAT module) and the C2f module to construct a DAC2f (DAttention-C2f) structure. Through a closed-loop design of precise extraction, efficient fusion, and high-quality representation, it adaptively captures the core features of occluded passengers, suppresses background interferences such as seat armrests, and breaks through the global fixed paradigm.

(1) DAT Module

To address bus scenarios’ enclosed space, high passenger density, and frequent occlusions, DAttention is specifically designed to tackle occlusions: it generates unified grid reference points and dynamic offset vectors via a lightweight sub-network, focusing on key target regions; it uses bi-linear interpolation for feature sampling at deformed points to capture incomplete features of occluded passengers; and it incorporates a deformable relative positional bias to enhance spatial dependency modeling, facilitating differentiation between occluded targets and background interferences.

The core logic of the DAT Module lies in the accurate capture of key features through dynamic sampling, with the following specific process: First, a unified grid reference point set is generated for the input feature map $P\in R^{H_{C}\times W_{G}\times 2}$. The grid size is determined by the downsampling factor r, $H_{G}=H/r,W_{G}=W/r$, The coordinates of the reference points are normalized to the range [0, 1], where (0, 0) denotes the top-left corner and (1, 1) denotes the bottom-right corner.

Subsequently, the feature map is linearly projected into a dimension-consistent query vector and key-value feature pairs for the subsequent attention calculation query token $q=xW_{a}$, that is input into the lightweight sub-network $\theta_{\mathrm{of}\;\mathrm{fest}(.)}$ to generate the offset vectors $\Delta_{P}=\theta_{\mathrm{of}\;\mathrm{fest}(.)}$. A predefined factor is introduced to constrain the amplitude of the offset vectors, ensuring training stability. Feature sampling is performed at the deformed point positions to construct keys and values. The bi-linear interpolation method is adopted during the sampling process to guarantee differentiability, and the definition of the sampling function is given in Equation (1):

(1) $$\phi \left(z;\left(p_{x},p_{y}\right)\right)=\sum_{\left(r_{x},r_{y}\right)}g\left(p_{x},r_{x}\right)g\left(p_{y},r_{y}\right)z\left[r_{y},r_{x},:\right]$$

Among them, g(a,b)=max(0,1−|a−b|), only the 4 integral points near the target point are non-zero, enabling efficient local feature extraction. Finally, the output features are obtained through attention computation as shown in Equation (2):

(2) $$z^{\left(m\right)}=\sigma \left(q^{\left(m\right)}{\tilde{k}}^{(m)⊤}/\sqrt{d}+\phi \left(32B;R\right)\right){\tilde{v}}^{\left(m\right)}$$

(2) Offset Generation

**Figure 1:**
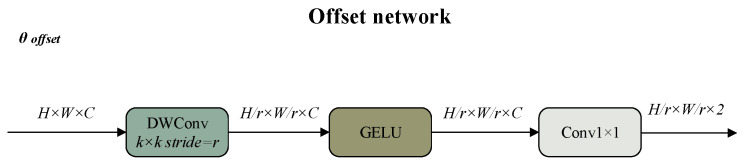
Offset network.

The core design of the offset generation sub-network is to enhance local feature perception for learning reasonable offsets, and its architecture is shown in Figure 1. The input features first capture local spatial information through a 5 × 5 depthwise convolution. After introducing non-linearity via the GELU activation function, a 1 × 1 convolution outputs 2D offset vectors. To avoid forced global offsets, the bias term of the 1 × 1 convolution is specifically reduced to ensure the adaptability and stability of the offset generation, while matching the characteristic that reference points cover a local s × s region.

(3) Offset Groups

To promote the diversity of deformed points, drawing on the grouping paradigm of Multi-Head Self-Attention (MHSA), the feature channels are divided into G groups. Each group of features independently generates offset vectors through a shared sub-network. In practical deployment, the number of heads (M) of the attention module is set as an integer multiple of the offset groups (G), ensuring that multiple attention heads can collaboratively adapt to each group of deformed keys and values, thereby enhancing the richness of feature representation.

(4) Deformable Relative Position Bias

To strengthen the spatial information encoding capability, a deformable relative position bias mechanism is introduced: for a feature map of size H×W, the range of its relative coordinate displacements is [−H,H] and [−W,W]. Different from the bias table based on discrete displacements in the Swin Transformer $\hat{B}\in R^{\left(2H-1)\times (2W-1\right)}$, this paper normalizes the relative displacements to the range [−1,+1] and performs interpolation in a continuous relative bias table $\phi (\hat{B},R)$. This achieves full coverage of all possible offset values and strengthens the modeling of spatial dependencies between queries and keys.

(5) Model Architecture

**Figure 2:**
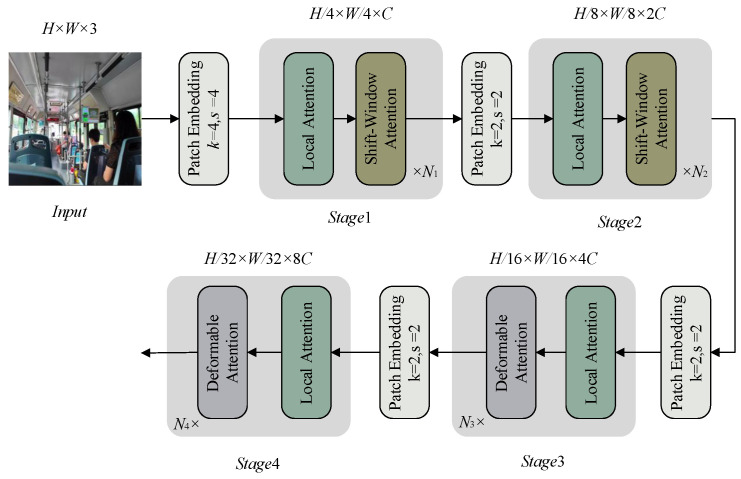
DAttention model architecture.

DAttention adopts a pyramid structure suitable for multi-scale visual tasks, as shown in Figure 2. The input image undergoes non-overlapping convolutional embedding and normalization to obtain patch embedding features of size (H×W×3). After non-overlapping convolutional embedding and normalization with a kernel size of 4×4, patch embedding features of size H/4×W/4×C are obtained. The backbone network consists of 4 stages, realizing hierarchical feature extraction through increasing stride. Between stages, 2×2 on-overlapping convolution (stride = 2) is adopted for downsampling, which halves the spatial size and doubles the feature dimension, ultimately constructing a complete multi-scale feature pyramid.

#### 3.1.2. SWD-PAN Structure

**Figure 3:**
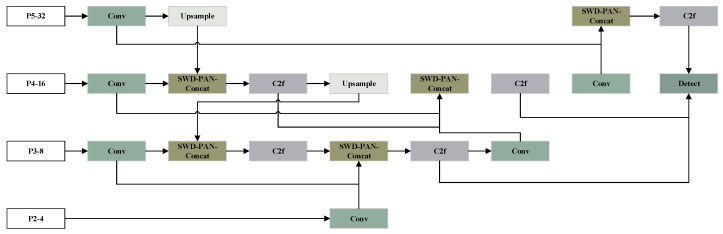
SWD-PAN network architecture.

To address the unidirectional information flow, equalized feature weight allocation, and low efficiency of cross-scale connections in traditional Feature Pyramid Networks (FPN), this paper proposes the SWD-PAN (Slim Weighted Dynamic Path Aggregation Network) bidirectional feature pyramid network. It achieves triple optimizations: bidirectional flow, weighted adaptation, and structural simplification, constructing an efficient feature fusion architecture that enables bidirectional cross-scale feature interaction and adapts to scale differences, thus enhancing target detection accuracy in dense scenarios. Its network architecture is illustrated in Figure 3.

(1) Bidirectional Feature Fusion Mechanism

A top-down and bottom-up bidirectional flow path is established to achieve iterative fusion of high-level semantic features (suitable for large-scale targets) and low-level detailed features (suitable for small-scale targets), which is distinct from the unidirectional FPN that fails to fully integrate cross-scale information.

(2) Learnable Weighted Fusion

Dynamic weights are assigned to features of different scales to highlight the contribution of scale-adaptive features (e.g., higher weights are allocated to small-scale features when passengers are far from the camera), addressing the issue of equal weight distribution in traditional fusion. Its core formula is as follows in Equation (3):

(3) $$O=\alpha \sum_{i=1}^{n}w_{i}\cdot I_{i}^{\mathrm{top}-\mathrm{down}}+\left(1-\alpha \right)\cdot \sum_{j=1}^{m}w_{j}\cdot I_{j}^{\mathrm{bottom}-\mathrm{up}}$$

where α is the bidirectional flow balance coefficient, with a range of [0, 1]. $w_{i}/w_{j}$ is the scale-adaptive weight. $I_{i}^{\mathrm{top}-\mathrm{down}}/I_{j}^{\mathrm{bottom}-\mathrm{up}}$ are the features of the top-down and bottom-up paths, respectively. This formula embodies bidirectional transmission, weighted adaptation, and multi-scale integration, consistent with the structural design of SWD-PAN.

(3) Structural Optimization Strategies

Add direct connections at the same layer to retain local scale features, and reuse bidirectional paths for multi-round iteration, further enhancing the network’s adaptability to rapid scale changes.

#### 3.1.3. WIoU Loss Function

The design of the bounding box loss function directly impacts detection box localization accuracy in target detection. This paper adopts the WIoU loss function, which uses a dynamic non-monotonic focusing mechanism to reduce the training weight of extreme samples with large gradients and increase that of medium-quality anchor boxes, avoiding gradient oscillation and accelerating stable convergence. It also dynamically adjusts gradient gain based on IoU value, making the loss function more sensitive to small positional errors of passenger targets, thus speeding up bounding box localization convergence and meeting detection requirements for small targets and passengers in non-standard postures in bus scenarios.

The penalty term formulas of the representative EIoU, Focal-EIoU (F-EIoU), and WIoU are as follows in Equations (4)–(6):

(4) $$\mathrm{Loss}\left(IoU\right)=1-\frac{\left|A\cap B\right|}{\left|A\cup B\right|}$$

(5) $$\mathrm{Loss}\left(EIoU\right)=1-IoU+\frac{\rho^{2}\left(b,b^{gt}\right)}{{\left(w^{c}\right)}^{2}+{\left(h^{c}\right)}^{2}}+\frac{\rho^{2}\left(w,w^{gt}\right)}{{\left(w^{c}\right)}^{2}}+\frac{\rho^{2}\left(h,h^{gt}\right)}{{\left(h^{c}\right)}^{2}}$$

(6) $$\;\mathrm{Loss}\;\left(F-EIoU\right)=IoU^{\gamma }\;\mathrm{Loss}\;\left(EIoU\right)$$

For a given predicted box *B* and ground-truth box $B^{gt}$, *b* and $b^{gt}$ denote the center points of *B* and $B^{gt}$; *w* and $w^{gt}$ represent the widths of *B* and $B^{gt}$; and *h* and $h^{gt}$ represent the heights of *B* and $B^{gt}$. $\rho \left(\cdot \right)={∥b-b^{gt}∥}_{2}$ denotes the Euclidean distance. *c* is the diagonal length of the minimum enclosing rectangle (MER) covering both boxes. $w^{c}$ and $h^{c}$ are the width and height of the MER covering both boxes. γ denotes the parameter controlling the consistency of outliers. They are as follows in Equations (7)–(11):

(7) $$\mathrm{Loss}\left(WIoUv1\right)=R_{WIoU}L_{IoU}$$

(8) $$R_{\mathrm{WIoU}\;}=\exp\frac{{\left(b_{c_{x}}^{gt}-b_{c_{x}}\right)}^{2}+{\left(b_{c_{y}}^{gt}-b_{c_{y}}\right)}^{2}}{{\left({c_{w}}^{2}+{c_{h}}^{2}\right)}^{*}}$$

(9) $$\mathrm{Loss}\left(W\;\mathrm{IoU}\cup 2\right)=\mathrm{loU}U^{\gamma }\mathrm{Loss}\left(W\;\mathrm{IoU}\cup 1\right)$$

(10) Loss(W IoU∪3)=Loss(W IoU∪1)×r

(11) $$r=\frac{\beta }{\delta \alpha^{\beta -\delta }},\beta =\frac{IoU^{\gamma }}{IoU}$$

Among them, $b_{c_{x}}^{\mathrm{gt}}$ and $b_{c_{y}}^{gt}$ denote the coordinates of the center point of the ground-truth bounding box; $b_{c_{x}}$ and $b_{c_{y}}$ denote the coordinates of the center point of the predicted bounding box. $c_{w}$ represents the width difference between the predicted bounding box and the ground-truth bounding box; $c_{h}$ represents the height difference between the predicted bounding box and the ground-truth bounding box; α=1.9; δ=3; and “*” denotes an operation that separates $c_{w}$ and $c_{h}$ from the computation process to prevent R from hindering the convergence speed. The advantages and disadvantages of the above partial loss functions are compared in Table 1.

In bus scenarios, the design of the loss function is crucial to the model’s detection performance and is attributed to the high proportion of small targets and significant differences in target scales. WIoUv3 adopts a dynamic non-monotonic mechanism to evaluate anchor box quality and designs a reasonable gradient gain allocation strategy, reducing the occurrence of large gradients or harmful gradients in extreme samples. On this basis, WIoUv3 focuses more on anchor boxes with average quality to improve the model’s recognition accuracy and generalization ability.

To verify the adaptability of each loss function, comparative experiments are conducted on a mixed dataset consisting of public datasets and a self-built bus-scenario dataset: 10,000 images are randomly selected. The experiments are performed in an environment with CPU (Intel (R) Core (TM) i7-14700HX), GPU (NVIDIA GeForce RTX 4060), 16 GB memory, and Python 3.9.19 + PyTorch 2.3.1 (GPU). With 150 training epochs as the standard, the detection performance of different loss functions is compared (as shown in Table 2).

As can be seen from the data in Table 1 and Table 2, the WIoUv3 loss function achieves the optimal performance: its precision reaches 86.3%, which is 17.41% higher than that of SIoU and 2.49% higher than that of GIoU; the mAP50 reaches 74.2%, which is 4.80% higher than that of the basic IoU and 0.27% higher than that of SIoU. Meanwhile, both the recall rate (63.1%) and frame rate (460 FPS) can meet the real-time application requirements of bus scenarios. Its core advantages lie in: reducing the gradient interference of extreme samples through the dynamic non-monotonic focusing mechanism, strengthening the training weight of anchor boxes with medium quality, effectively improving the localization accuracy of detection boxes for passengers with non-standard postures, and perfectly adapting to the complex detection needs of bus scenarios.

**Figure 4:**
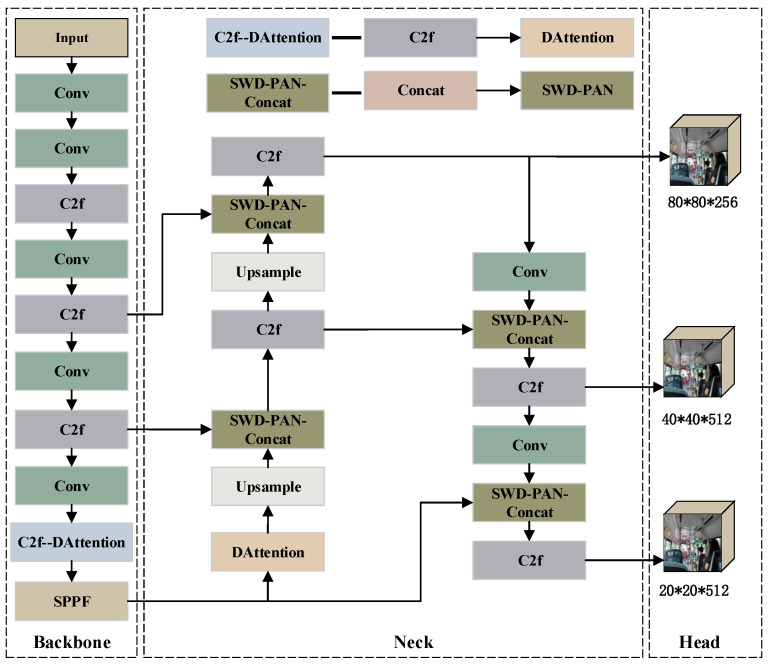
Improvement of the YOLOv8 network structure diagram.

The architecture of the improved YOLOv8 network is shown in Figure 4. The core achieves a leap in bus passenger object detection performance through the full-link collaborative design of “feature extraction-feature fusion-loss optimization”: the DAttention mechanism is embedded in the Backbone and Neck modules, which are deeply integrated with the C2f module to construct the DAC2f structure; the Neck module adopts the SWD-PAN network to enhance multi-scale feature fusion; and the WIoUv3 loss function is introduced into the entire network to optimize detection box localization.

### 3.2. Tracker

To address the tracking challenges brought by multi-scale, high-occlusion, non-standard postures, and the dynamic movement of passenger targets in complex bus scenarios, this paper proposes the DA-SWD-YOLOv8+DeepSORT multi-object detection and tracking algorithm. By deeply coupling high-precision object detection features with a robust motion–appearance association mechanism, it realizes continuous and stable tracking of passenger targets and accurate passenger flow statistics. Its core design and complete process are as follows.

#### 3.2.1. Core Design of the Algorithm

(1) Enhancement of Feature Extraction in the Detection Module

To address the problem of insufficient detection accuracy of general-purpose detectors in bus scenarios with high density and occlusion, the DAttention deformable attention mechanism is fused with the C2f module to construct the DAC2f structure. It dynamically samples key regions like passengers’ heads and trunks, generates adaptive offset vectors to capture core features while suppressing background interferences, embeds the GELU activation function and lightweight offset generation sub-network to balance feature capture flexibility and gradient propagation stability, and avoids gradient vanishing or oscillation during training. The SWD-PAN weighted bidirectional feature pyramid network is introduced at the neck network level, which adopts bidirectional feature flow and learnable weight allocation to prioritize enhancing the feature weights of small-sized and occluded passengers.

(2) Optimization of the Detection Model Training Mechanism

The WIoUv3 loss function is adopted to accurately evaluate anchor box quality via a dynamic non-monotonic focusing mechanism, optimizing non-standard posture passenger anchor box fitting. Combined with the gradient gain allocation strategy, it balances extreme and medium-quality sample weights, reducing invalid gradient interference and enhancing detection box localization accuracy in the improved YOLOv8n. At the training target level, bounding box localization, classification, and confidence losses are comprehensively optimized, with network parameters iteratively updated via backpropagation and real-time detection performance evaluated using the validation set to form a “training-validation-weight optimization” closed loop for the improved YOLOv8n adapted to bus scenarios.

(3) Deep Integration of Detection-Tracking Modules

High-quality detection results filtered by a confidence threshold (conf-thres = 0.05) are input into the DeepSORT tracker. The BoT network enhances the appearance feature extraction module to capture discriminative information like clothing textures and local postures, reducing ID switching in occluded scenarios. The Kalman filter adjusts parameters for short-distance, low-speed bus passenger movements, efficiently coupling detection and tracking with highly discriminative appearance features.

#### 3.2.2. Improved Algorithm Flow

The process consists of two phases: Phase 1 (Model Training and Optimization Closed Loop) and Phase 2 (Tracking Inference and Result Output). Phase 1 starts with constructing a dedicated bus passenger detection dataset—completing refined target annotation and splitting it into training/validation sets at a reasonable ratio for high-quality data support. Next, core training parameters (epochs = 250, conf-thres = 0.05, iou-thres = 0.6, batch-size = 32, alpha = 3 in Alpha-IoU) are set, and the model structure is improved by embedding the DAC2f structure, integrating the SWD-PAN feature fusion network, and replacing CIoU with WIoUv3 loss. After loading the network structure, parameters, and dataset, iterative training is conducted to extract passenger features and optimize localization accuracy; the sum of WIoUv3 localization loss, classification loss, and confidence loss is calculated in real time, with network parameters updated via backpropagation. After each epoch, the validation set evaluates performance—updating suboptimal weights and saving the optimal model after 250 epochs to output a bus-scenario-adapted high-precision detector. Phase 2 inputs the optimal model’s detection results into DeepSORT, filtering overlapping boxes via NMS to retain optimal target boxes. The tracker judges historical trajectory matches: if matched, Kalman filtering (kf.predict (self.mean, self.convariance)) predicts the current frame’s candidate position; if not, it marks unmatched detections/tracks. Through cascade and IoU matching, successfully matched trajectories are updated via track.update, unmatched detections get new trajectories via -initiate-track (detections), and unmatched tracks are marked via track.mark-missed (ID retained/deleted based on motion characteristics). Finally, passenger target bounding box coordinates, unique IDs, and relevant attributes are output, providing continuous reliable tracking data for subsequent bus passenger flow statistics.

### 3.3. Bus Passenger Boarding/Alighting Counting and Extraction Algorithm

To identify passengers in boarding or alighting states, a detection line is set to count such passengers in videos. The process involves judging target behavior, determining boarding/alighting, counting passengers, and generating a corresponding dataset.

Step 1: Input monitoring videos of passengers boarding and alighting. Step 2: The improved YOLOv8 model detects passenger targets in each video frame, returning their positions and extracting features. Step 3: Input the position and feature data into the DeepSORT model for trajectory matching to get unique IDs. Step 4: Set line-crossing points on the tracking bounding boxes. Step 5: The Detector class in objdetector.py encapsulates the YOLOv8 detector and instantiates it. Step 6: Call the Tracker.update method of DeepSORT to get video frames and passenger bounding boxes. Step 7: Judge if the passenger’s trajectory is boarding/alighting behavior, and if so, check line-crossing. Step 8: Judge line-crossing and count passengers if applicable, else delete the ID. Step 9: Update the counter to realize passenger flow statistics.

## 4. Experiment and Result Analysis

### 4.1. Experimental Conditions and Datasets

#### 4.1.1. Experimental Conditions

The hardware and software environments of this experiment are as follows: Hardware: CPU (Intel(R) Core(TM) i7-14700HX), GPU (NVIDIA GeForce RTX 4060), 16 GB memory; Software: Windows OS, Python 3.9.19, PyTorch 2.3.1 (GPU version).

#### 4.1.2. Dataset

(1) Optimization of Bus Passenger Target Detection Dataset

To better train the model, the training dataset is expanded. Searching on the Roboflow platform with “Passenger” as the keyword yields datasets of passengers in buses, including: bus passenger detection, human head in bus, human head detection, passenger head, and head detection. These datasets are exported in the .txt format required for YOLOv8 model training, resulting in 11,967 training images and 1811 validation images.

The self-made dataset in this paper was collected from real bus interior scenarios and captured using a DJI Pocket 3 handheld camera. The shooting scenarios include passenger boarding and alighting at the front and rear doors, as well as passenger data inside the vehicle. Five complete bus routes were collected: four full videos of front/rear door passenger boarding/alighting from start to terminal stations and one partial rear-to-front video, with a total duration of about 5 h 40 min. Images were extracted every 240 frames (empty frames excluded), yielding 17,492 images, which were randomly split into a 14,827-image training set and a 2665-image validation set. All images were annotated via labelImg: rectangular boxes were drawn around passengers’ full bodies as model detection targets, directly generating the .txt files for model training.

**Figure 5:**
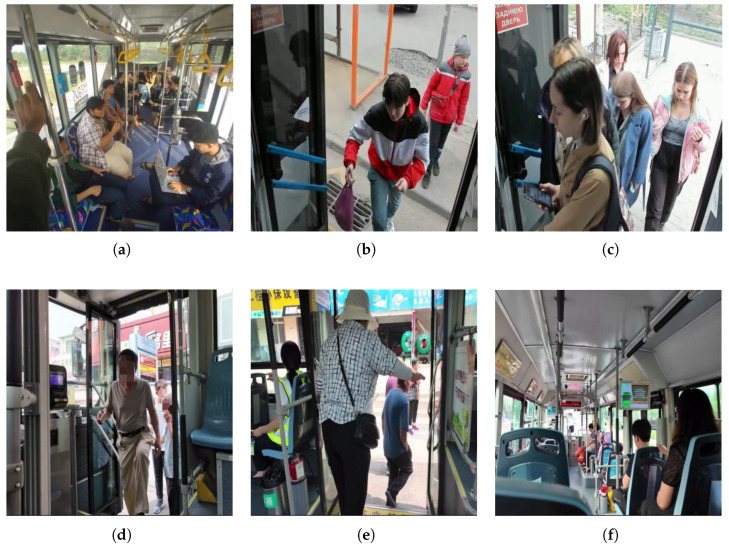
Selected image data within the dataset (**a**) In-vehicle image (**b**) Boarding image (**c**) Boarding image (**d**) Boarding image (**e**) Alighting image (**f**) In-vehicle image.

The public dataset and self-made dataset were merged to form a total of 26,794 training images and 4476 validation images. The test set, consisting of 3127 images, was further divided from the combined dataset in a 7:2:1 ratio, which collectively constitutes the passenger object detection dataset to ensure the independence and representativeness of performance evaluation.As shown in Figure 5.

(2) Target Tracking and Passenger Boarding/Alighting Extraction

**Figure 6:**
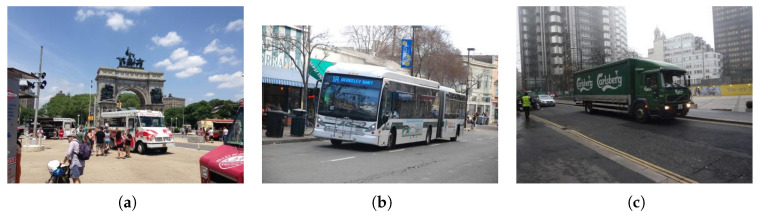
COCO dataset.

The COCO128 dataset, a lightweight subset of COCO, is designed for rapid prototyping and algorithm validation in object detection, being easier to handle than the original. It includes 128 annotated images across 80 object categories (humans, animals, vehicles, etc.), with 2D bounding boxes for multiple targets per image—some also have segmentation and keypoint annotations in COCO JSON format (category, coordinates, contours, etc.). Despite its small size, it retains the original dataset’s diversity and complexity (varying lighting, occlusion, overlap), posing training/test challenges, and is widely used for initial algorithm development to enable rapid iteration and evaluation, as shown in Figure 6.

**Figure 7:**
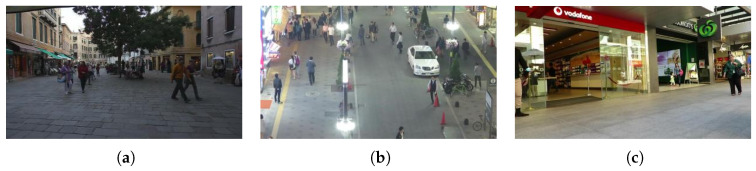
MOT-17 dataset.

The MOT-17 dataset, a key Multiple-Object Tracking (MOT) Challenge benchmark for pedestrian tracking, includes seven training and seven test sequences focusing on complex urban pedestrian tracking, with frequent pedestrian interaction, occlusion, overlap and diverse trajectories. Each frame is annotated with pedestrian 2D bounding boxes, unique IDs, and occlusion/truncation info for evaluating algorithm identity preservation, split into training/validation/test sets at a ratio of 7:2:1. Its core challenge is complex occlusions demanding high algorithm robustness, with targets concentrated on pedestrians. MOT-17 subsets vary in scenarios and sensor collection: Subset 02 is low-density/low-occlusion via a fixed camera, while Subset 04 is high-density/high-occlusion via a mobile platform camera, matching bus on-board sensor features and serving as the key subset for verifying the proposed algorithm’s complex scenario adaptability [36,37,38], as shown in Figure 7.

**Figure 8:**
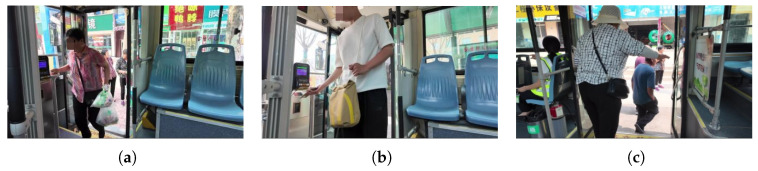
Self-Made Dataset.

The self-built dataset is collected via a DJI Pocket 3 camera from real-bus interior scenarios, including passenger boarding/alighting at front/rear doors and in-vehicle passenger data, applicable for passenger detection, tracking and boarding/alighting counting, as shown in Figure 8.

### 4.2. Evaluation Metrics

#### 4.2.1. Object Detection and Discrimination

Six evaluation metrics are adopted for the network model: precision (P), recall (R), average precision (AP), frames per second (FPS), F1-score, and mean average precision (mAP). Among them, F1-score and AP are correlated with precision and recall, and their calculation formulas are as follows in Equations (12)–(16):

(12) $$\;\mathrm{Precision}\;=\frac{TP}{TP+FP}$$

(13) $$\;\mathrm{Recall}\;=\frac{TP}{TP+FN}$$

(14) $$AP=\int_{0}^{1}P\left(R\right)dR$$

(15) $$\;F1-\mathrm{score}\;=\frac{2\times P\times R}{P+R}$$

(16) $$mAP=\frac{1}{N}\sum_{i=1}^{N}AP_{i}$$

where *TP* (true positives) refers to the number of positive samples correctly identified; *FP* (false positives) refers to the number of positive samples incorrectly identified; *FN* (false negatives) refers to the number of negative samples incorrectly identified.

#### 4.2.2. Target Tracking and Discrimination of Passenger Boarding/Alighting

(1) MOTA

MOTA (Multiple-Object Tracking Accuracy) is a comprehensive evaluation metric for multi-object tracking, used to improve the accuracy of multi-object tracking. It leverages the matching degree between the trajectories output by the tracker and the ground-truth trajectories for large-scale data training, thereby enhancing the discrimination accuracy of the tracker. Its calculation formula is as follows in Equation (17):

(17) $$MOTA=1-\frac{FN+FP+IDsw}{GT}$$

where *FN* = missed ground-truth targets; *FP* = incorrectly identified non-targets; *IDsw* = same-target ID changes across frames; and *GT* = total ground-truth targets. *MOTA* accounts for misses, false detections and ID switches—higher values mean better tracker performance.

(2) MOTP

MOTP (Multiple-Object Tracking Precision) is another important evaluation metric, used to measure the average error between the positions predicted by the tracker and the ground-truth positions, and to describe the tracking precision of multiple objects. MOTP calculates the average of the position errors of all correctly matched targets output by the tracker. Its calculation formula is as follows in Equation (18):

(18) $$MOTP=\frac{\sum_{i=1}^{N}\sum_{t=1}^{T}\mathrm{dist}\left(i,t\right)}{{\mathrm{Total}}_{\mathrm{correct}\;}}$$

where *N* = correctly matched targets across all frames, *T* = number of frames, dist(*i*,*t*) = position estimate of ground-truth distance (e.g., Euclidean) of the *i*-th target in frame *t*, and Totalcorrect = total correctly matched targets. Higher *MOTP* means more accurate position prediction; it pairs with MOTA (evaluates detection/ID assignment performance) to assess trackers.

(3) IDF1

IDF1 is an evaluation metric in Multi-Object Tracking (MOT), which measures the accuracy and consistency of the tracker in identifying target identities. IDF1 introduces the concepts of precision and recall: precision measures the tracker’s ability to correctly identify target identities, while recall measures the tracker’s ability to cover all ground-truth targets. Its calculation formula is as follows in Equation (19):

(19) $$IDF1=\frac{2\times IDP\times IDR}{IDP+IDR}$$

where *IDP* (ID Precision) is the target identity precision, representing the ratio of the number of correctly identified targets to the total number of identified targets; *IDR* (ID Recall) is the target identity recall, representing the ratio of the number of correctly identified targets to the total number of ground-truth targets. A higher value indicates better performance of the tracker in target identity identification.

(4) HOTA

HOTA (Higher-Order Tracking Accuracy) is a metric for comprehensively evaluating multi-object tracking performance, which mainly focuses on multiple factors in detection and tracking tasks, including position, ID, visual appearance, etc. The calculation of HOTA involves the combination of multiple sub-metrics, including three main components: Localization HOTA (LHOTA), Identity HOTA (IHOTA), and Detected HOTA (DHOTA). Its calculation formula is as follows in Equation (20):

(20) HOTA=LHOTA×IHOTA×DHOTA

(5) AssA

AssA (Association Accuracy) is a metric for measuring the performance of the tracker in terms of association correctness. AssA measures accuracy by calculating the matching degree between the associations predicted by the tracker and the ground-truth associations.

(6) DetA

DetA (Detection Accuracy) is a metric for measuring the accuracy of the tracker in detection tasks, i.e., the detection accuracy of the detector. DetA measures the degree of correct target detection by the tracker, calculated as the ratio of the number of targets correctly detected by the tracker to the total number of ground-truth targets.

### 4.3. Experiments on Object Detection Optimization

#### 4.3.1. Ablation and Comparison Experiments on YOLOv8

To verify the impact of the improved modules on the overall network performance, ablation and comparison experiments are conducted in this paper, and the experimental results are shown in Table 3. The network with DAttention added has stronger feature extraction capability: compared with the YOLOv8n network, the precision (P) value is increased by 1.54%, the recall (R) value is increased by 1.79%, and the mAP value is increased by 1.17%. By introducing the weighted bidirectional feature pyramid structure, the P value is increased by 0.82%, the R value is increased by 1.38%, and the mAP value is increased by 1.04%. Compared with the previous network configuration, the network with the improved loss function has a 1.28% increase in P value, a 1.86% increase in R value, and a 1.48% increase in mAP value.

In this paper, the improved YOLOv8 algorithm is compared with several currently popular classic algorithms, namely YOLOv5n, SSD, Faster-RCNN, and YOLOv8n (as shown in Table 4). The precision (P) value of the improved algorithm is increased by 4.55%, 4.81%, 4.43%, and 3.68% compared with these four algorithms, respectively; the recall (R) value is increased by 4.99%, 8.45%, 2.24%, and 5.12%, respectively; the mAP50 value is increased by 4.71%, 5.45%, 3.01%, and 3.73%, respectively; and the mAP50-95 value is increased by 8.17%, 1.36%, 7.12%, and 6.26%, respectively. The FPS (frames per second) value decreases significantly due to the integration of the DAttention mechanism, but it still meets the requirements of practical applications.

**Figure 9:**
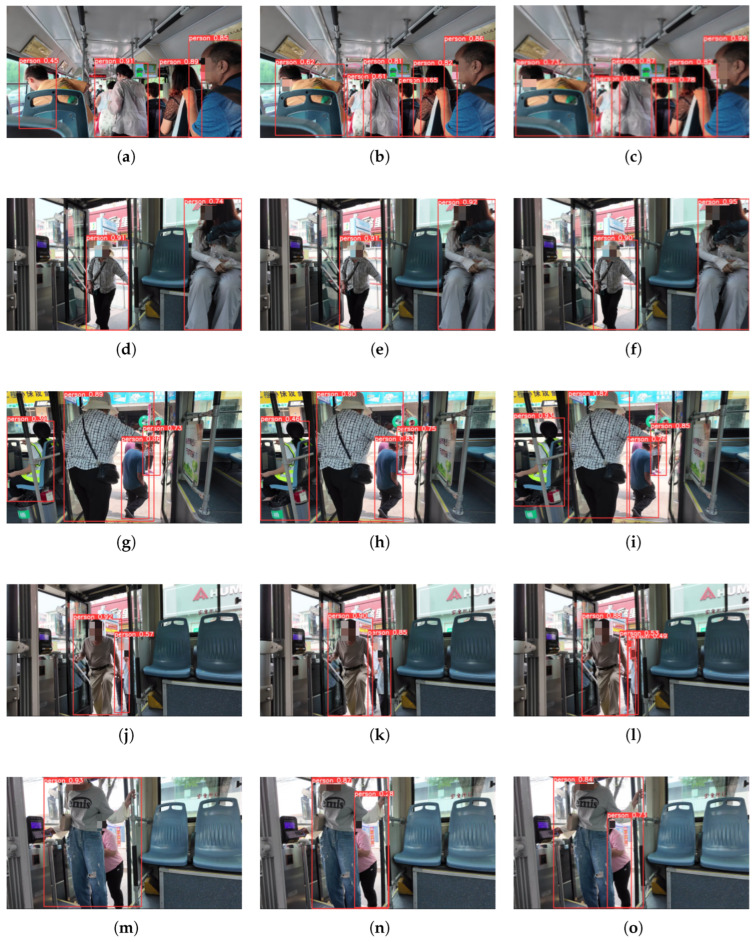
Chart of predicted results (Column 1: YOLOv5n, Column 2: YOLOv8n, Column 3: YOLOv8-g).

The improved model is tested in real-world scenarios against YOLOv5n and YOLOv8n, as shown in the comparisons of Figure 9. The improved YOLOv8 network significantly enhances its adaptability to targets from different perspectives and anti-interference capability in occluded scenarios, and can stably accomplish the accurate detection task of passenger targets. In the target-dense scenario shown in Figure 9, compared with YOLOv5n and YOLOv8n, the algorithm not only effectively captures more potential targets (only three pedestrian targets are successfully identified in Figure 9a, while six pedestrian targets are successfully identified in Figure 9c), with a substantial improvement in detection coverage, but also further reduces the probability of false detection and missed detection, achieving dual optimization of detection quantity and accuracy, which fully meets the passenger detection requirements in complex scenarios.

The improved YOLOv8 network has better recognition capability in the face of targets from different perspectives and the adverse effects of occlusion, and can smoothly complete the detection task of target passengers.

To sum up, the improved YOLOv8 algorithm for bus passenger target detection can accomplish the static detection and recognition of bus passenger targets, and the next research direction is the dynamic tracking of bus passenger targets.

#### 4.3.2. Statistical Significance Verification

To verify the reliability of the performance improvement of the improved algorithm, a *t*-test was used to conduct a statistical significance analysis on the key metrics of the model proposed in this study and the baseline YOLOv8. The confidence level was set at 95% (α = 0.05). Each algorithm was independently run 5 times, and the values of each metric were recorded. The results are shown in Table 5.

The improved model’s *p*-values for three key metrics are all <0.05, proving the performance improvement is a significant algorithm-driven effect rather than random fluctuations. The smallest mAP50 *p*-value verifies the statistical reliability of SWD-PAN and WIoUv3 fusion for boosting detection accuracy.

#### 4.3.3. Result Analysis

The improved algorithm’s performance gain stems from the synergy of feature extraction, fusion and loss optimization: DAC2f boosts recall by 5.12% for dense targets via dynamic key-region sampling, SWD-PAN raises mAP50 by 3.73% through bidirectional weighted feature fusion, and WIoUv3 increases precision by 3.68% by optimizing non-standard posture target localization. Outperforming YOLOv5n and SSD, it achieves the most notable mAP50-95 improvement, excelling in small-target detection and localization critical for bus scenarios; its FPS drops to 356 yet meets on-board real-time demands, balancing accuracy and speed well.

### 4.4. Experiments on Target Tracking and Passenger Boarding/Alighting Extraction

#### 4.4.1. Trajectory Analysis of Bus Boarding and Alighting Passengers

To distinguish different states and behaviors in bus videos—such as passengers versus pedestrians and passenger boarding versus alighting—this paper extracts, tracks, and detects passenger trajectories while classifying them into four states: boarding state, alighting state, riding state, and waiting/pedestrian state. Passenger objects boarding or alighting during bus stops are extracted from the passengers’ (OD) information. Combined with the differentiation rules for passenger movement trajectories under different states, the trajectory information of boarding and alighting passenger objects is thus obtained.

**Figure 10:**
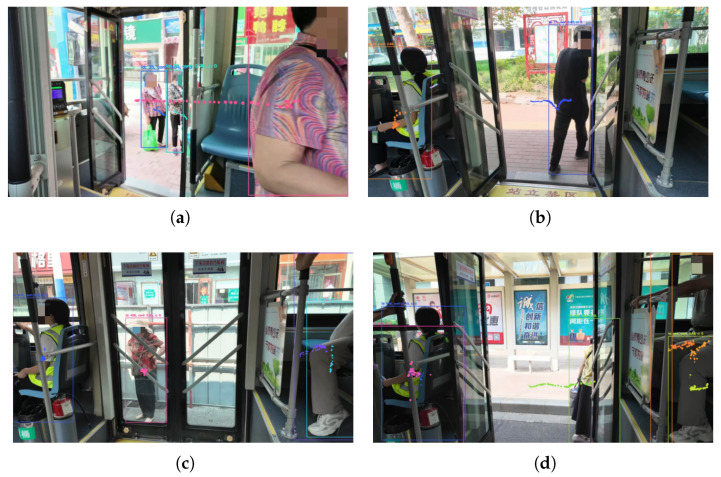
Public transport passenger trajectory map (**a**) Boarding State Trajectory (**b**) Alighting State Trajectory (**c**) Riding State Trajectory (**d**) Pedestrian State Trajectory.

The figure in Figure 10 shows four common movement trajectories of bus passengers, which are obtained by connecting the midpoints of the bottom lines of detection boxes in consecutive frames. The first figure shows a passenger in the boarding state: the trajectory starts outside the bus door and extends into the door. Image feature: taking the bottom of the door as the reference line, the trajectory will intersect the reference line from top to bottom. The second figure shows a passenger in the alighting state: the trajectory also intersects the reference line, but in the opposite direction. The third figure shows a passenger in the riding state: the passenger is relatively stationary, and the movement trajectory is relatively concentrated. The fourth figure shows a pedestrian: the trajectory demonstrates the pedestrian walking from left to right, with no moment of intersection with the reference line. To sum up, detection objects in different states exhibit different interaction processes between their movement trajectories and the reference line.

#### 4.4.2. DeepSORT Pedestrian Tracking Experiments

Experiments combining different detectors with the DeepSORT algorithm are conducted in this paper. The experimental results are shown in Table 6.

The improved method shows disparate performance gains on MOT-17 Subsets 02 (0.166% precision rise) and 04 (1.44% precision rise), rooted in their distinct scene complexity, target traits and sensor conditions. Subset 02 is an ideal low-density, low-occlusion scenario with fixed CMOS cameras, approximately 1.2 people/m^2^, ≤20% occlusion and no obvious interference; the baseline YOLOv8+DeepSORT performs near its upper limit here, leaving limited room for the improved modules to boost performance with minor total contribution. By contrast, Subset 04 is a high-density, high-occlusion dynamic scenario matching bus scene complexity, with mobile platform HD cameras, up to 3.5 people/m^2^, ≥40% occlusion and interference from rapid movement/vibration, where the baseline exposes flawed feature extraction/fusion. The improved DAC2f’s dynamic sampling captures occluded target core features and reduces background interference, SWD-PAN’s bidirectional weighted fusion strengthens multi-scale feature interaction, and anti-blur logic enhances cross-frame feature consistency. Statistical analysis of scene indicators and module contribution rates reveals small individual and total contributions of improved modules in low-complexity Subset 02, while DAC2f contributes over 50% to performance gain in high-complexity Subset 04 as the core driver. This disparity verifies the algorithm’s original design intent: adapting to high-density, high-occlusion, dynamic complex scenarios like bus boarding/alighting instead of all general scenarios. Bus scenarios align with Subset 04 in target density, occlusion and dynamics, plus extra sensor vibration/lighting interference; the significant gain on Subset 04 confirms its adaptability to bus scenarios, while the slight gain on Subset 02 is only marginal and does not undermine its core value for target scenarios.

**Figure 11:**
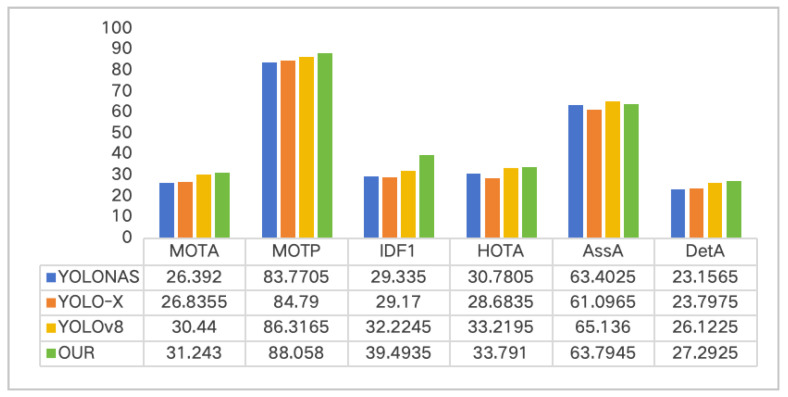
Combined results of detector experimental data.

The experimental results of various detection algorithms in Table 6 across different datasets of MOT-17 are merged and presented in Figure 11.

From the experimental data, the following conclusions can be drawn:

For the MOTA metric: The accuracy of the DeepSORT algorithm with the proposed detector reaches 31.24, demonstrating its performance advantage in multi-object tracking tasks. The accuracy of the DeepSORT algorithm combined with YOLOv8 as the detector is 30.44. The DeepSORT algorithm with its built-in YOLO-X detector achieves an accuracy of 26.84; although its performance is quite good, it is 2.6% and 16.4% lower than the proposed algorithm, respectively.

For the HOTA metric: The accuracy of the DeepSORT algorithm with the proposed detector is 33.79, which is significantly higher than other algorithms, increasing by 9.8%, 17.8%, and 1.72% compared with the other three algorithms. This indicates that the proposed algorithm is significantly superior to other algorithms in terms of comprehensiveness when various factors are fully considered.

For the IDF1 metric: Which combines the accuracy of object detection and identification, the proposed algorithm also takes the lead with a score of 39.49, indicating its high accuracy in recognizing and identifying targets. Additionally, for the MOTP metric: The DeepSORT algorithm using the improved YOLOv8 as the detector outperforms other algorithms, increasing by 5.1%, 3.9%, and 2.0% compared with the other three algorithms, respectively.

This superior performance can be further supported by related theoretical research: Dong G et al. [39] proposed a universal moving object segmentation method by learning temporal distribution and spatial correlation. It extracts more accurate target contours and motion context information, which effectively alleviates tracking drift caused by occlusion and dynamic scale changes—consistent with the experimental phenomenon that our algorithm maintains stable tracking for occluded and small targets.

Overall, the proposed algorithm performs excellently in multiple metrics, especially in multi-object tracking accuracy, identification score, and high-order target tracking accuracy.

**Figure 12:**
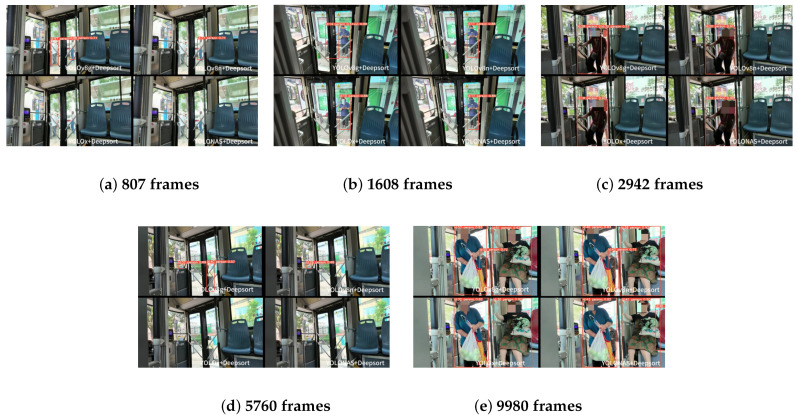
Partial tracking algorithm tracking effect diagram.

The figure in Figure 12 shows the tracking results at frames 807, 1608, 2942, 5760, and 9980, respectively. At frames 807 and 5760: the three detector algorithms (YOLOv8n, YOLO-X, and YOLONAS) all exhibit missed detections of small targets. At frame 9980: the YOLONAS algorithm shows a false detection. At frames 1608 and 2942: YOLOv8n, YOLO-X, and YOLONAS all fail to detect occluded targets (missed detections). In contrast, the proposed tracking algorithm in this paper can largely avoid missed detections and false detections. Meanwhile, it maintains good recognition and tracking performance even when the detected targets are occluded.

#### 4.4.3. Analysis of Passenger Boarding/Alighting Results

**Figure 13:**
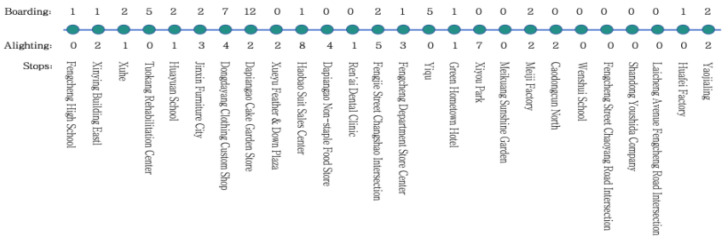
Map of bus stops and boarding/alighting numbers for Bus Route 3.

The experimental data uses a video shot on Bus Route 3 on 22 July 2025, covering the route from the starting station (Fengcheng High School) to the terminal station (Yaojialing). This route passes through a total of 26 stations, and its route map is shown in Figure 13.

For each bus stop, the video captured by the camera is clipped to obtain video data during the period from when the bus doors open to when they close. At each station, the number of boarding and alighting passengers through the front or rear door is counted. Manual counting is performed on the boarding and alighting passenger statistics collected from the front and rear door surveillance videos inside the bus, which is then compared with the results of the improved bus passenger target tracking algorithm and the original algorithm.

**Figure 14:**
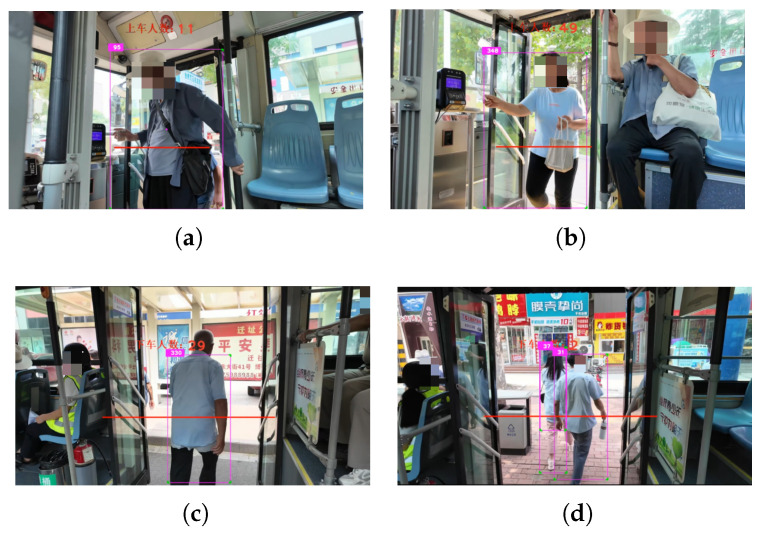
Public transport passenger flow statistics results (**a**) Boarding Passenger Flow Statistics (**b**) Boarding Passenger Flow Statistics (**c**) Alighting Passenger Flow Statistics (**d**) Alighting Passenger Flow Statistics.

As shown in Table 7, the proposed improved algorithm achieves higher statistical accuracy in both boarding and alighting scenarios than the original algorithm, reaching 98.1% and 95.9% respectively. The decrease in statistical accuracy is attributed to duplicate counting caused by some passengers alighting through the front door and missed counting due to partial passenger occlusion. Regarding the inconsistency between the number of boarding and alighting passengers, it may be because some passengers board in advance near the terminal station but do not alight at the terminal, leading to an imbalance between boarding and alighting numbers. The figure in Figure 14 shows a partial schematic diagram of the passenger flow statistics results.

## 5. Conclusions

To address bus scenarios’ core pain points (high-density, heavy-occlusion, and variable-target scales) and existing algorithms’ flaws (low accuracy, severe missed detections, and poor robustness), this paper designs improved YOLOv8-based detection and DeepSORT-based tracking algorithms, achieving over 98% accuracy in passenger boarding/alighting statistics via dataset training and on-site bus verification. The improved YOLOv8n fuses deformable attention with the C2f module to build the DAC2f structure for capturing occluded passengers’ core features and suppressing background interference, adopts the SWD-PAN network for cross-scale feature bidirectional interaction, and replaces CIoU with WIoUv3 for dynamic sample weight balancing. It outperforms the baseline YOLOv8n by 3.68% in precision, 5.12% in recall and 6.26% in mAP, maintains real-time speed, and boosts robustness in dense, occluded and multi-scale scenarios, reducing false and missed detections. The improved DeepSORT realizes accurate detection box state estimation via Kalman filtering, optimizes inter-frame matching by fusing appearance features, motion information and cascaded matching, and integrates with the improved YOLOv8; it introduces a trajectory discrimination method for boarding/alighting behaviors, achieving a 31.24% MOTA (2.6% and 16.4% higher than YOLOv8n and YOLO-X) and 88.06% MOTP, solving trajectory breakage and ID switching, and showing significant advantages in high-complexity bus-like scenarios despite limited gains in low-complexity ones. This study has limitations: hardware-dependent performance with reduced small-target detection accuracy in extreme dark environments, and insufficient dataset coverage (harsh weather, special groups) leading to poor adaptability. Future research will focus on hardware-algorithm collaboration and multi-sensor fusion for all-weather robustness, dataset expansion and semantic segmentation for optimized feature learning, non-linear filtering for better motion prediction, and model lightweighting for edge deployment, extending the algorithm to public transportation to support smart city transportation intellectualization.

## Figures and Tables

**Table 1:** Boundary loss performance comparison.

	mAP75	mAP50	mAP50-95
CIoU	53.03	63.14	45.20
SIoU	53.15	63.46	45.21
EIoU	53.55	63.17	45.39
F-EIoU	52.88	63.37	44.75
WIoU v1	52.82	63.15	44.87
WIoUv2 (γ = 0.5)	53.67	64.15	45.56
WIoU v3	54.50	64.20	64.20

**Table 2:** Loss function training performance comparison experiment.

Methods	Precision	Recall	mAP50	mAP50-95	FPS
YOLOv8	0.797	0.596	0.708	0.335	481
YOLOv8+GIoU	0.842	0.643	0.738	0.338	478
YOLOv8+DIoU	0.768	0.611	0.709	0.338	479
YOLOv8+CIoU	0.809	0.596	0.715	0.335	470
YOLOv8+SIoU	0.735	0.668	0.74	0.335	468
YOLOv8+EIoU	0.821	0.621	0.721	0.337	447
YOLOv8+PIoU	0.840	0.594	0.718	0.344	478
YOLOv8+F-EIoU	0.841	0.635	0.735	0.334	476
YOLOv8+WIoU v1	0.854	0.567	0.715	0.331	474
YOLOv8+WIoU v2	0.785	0.623	0.720	0.335	475
YOLOv8+WIoU v3	0.863	0.631	0.742	0.336	460

**Table 3:** Ablation experiment.

Method	Precision	Recall	mAP50	FPS
YOLOv8n	0.842	0.781	0.858	470
YOLOv8n+DAttention	0.855	0.795	0.868	446
YOLOv8n+DAttention+SWD-PAN	0.862	0.806	0.877	345
YOLOv8n+DAttention+SWD-PAN+WIoUv3	0.873	0.821	0.890	356

**Table 4:** Comparison of model evaluation indicators.

Method	Precision	Recall	mAP50	mAP50-95	Training Loss		
					Box_loss	Cls_loss	Dfl_loss
YOLOv5n	0.835	0.782	0.850	0.612	0.689	0.466	1.154
SSD	0.833	0.757	0.844	0.583	0.744	0.539	1.109
Faster R-CNN	0.836	0.803	0.864	0.618	0.7102	0.485	1.016
YOLOv8n	0.842	0.781	0.858	0.623	0.669	0.443	1.131
YOLOv8-g	0.873	0.821	0.890	0.662	0.312	0.453	1.161

**Table 5:** Statistical Significance Test Results.

Indicator	YOLOv8-g Mean ± Standard Deviation	YOLOv8n Mean ± Standard Deviation	t-Value	*p*-Value	Significance
Precision	0.873 ± 0.005	0.836 ± 0.008	8.62	0.002	significant
Recall	0.821 ± 0.007	0.770 ± 0.010	7.35	0.003	significant
mAP50	0.890 ± 0.006	0.853 ± 0.009	9.14	0.001	significant

**Table 6:** Experimental comparison results of different detectors+DeepSORT in MOT-17.

Detector	Dataset	MOTA	MOTP	IDF1	HOTA	AssA	DetA
YOLONAS	02	20.541	84.395	22.642	24.865	55.477	17.594
	04	32.243	83.146	36.028	36.696	71.328	28.719
YOLO-X	02	22.744	86.391	23.077	24.536	55.119	19.582
	04	30.927	83.189	35.263	32.831	67.074	28.013
YOLOv8	02	24.006	88.411	27.523	28.603	57.153	22.093
	04	36.874	84.222	36.926	37.836	73.119	32.152
OUR	02	24.172	88.656	31.222	28.566	56.995	21.209
	04	38.314	87.460	47.765	39.016	70.594	33.376

**Table 7:** Passenger boarding and alighting statistics.

Method	Number of Boarding Passengers	Number of Alighting Passengers
Manual counting	53	49
YOLOv8n+DeepSORT	50	45
YOLOv8n-g+DeepSORT	52	47

## Data Availability

The data used in this study includes COCO128 (accessed on 20 January 2025 and available at https://docs.ultralytics.com/datasets/detect/coco128/), MOT-17 (accessed on 20 January 2025 and available at https://motchallenge.net/data/MOT17/), and a self-built dataset. COCO128 and MOT-17 are provided in video and image formats, restricted to academic research/educational (COCO128) or non-commercial (MOT-17) use, requiring citation of original/related papers without modification or republishing; download via official website instructions. Public datasets can also be found by searching keywords on Roboflow (accessed on 20 January 2025 and available at https://universe.roboflow.com/). The self-built dataset, after desensitization for sensitive information, offers samples and annotation files for non-commercial research upon request to the authors.
